# Metabolic biomarkers of response to the AKT inhibitor MK-2206 in pre-clinical models of human colorectal and prostate carcinoma

**DOI:** 10.1038/s41416-018-0242-3

**Published:** 2018-10-31

**Authors:** Nada M. S. Al-Saffar, Helen Troy, Anne-Christine Wong Te Fong, Roberta Paravati, L. Elizabeth Jackson, Sharon Gowan, Jessica K. R. Boult, Simon P. Robinson, Suzanne A. Eccles, Timothy A. Yap, Martin O. Leach, Yuen-Li Chung

**Affiliations:** 1 Cancer Research UK Cancer Imaging Centre, Division of Radiotherapy and Imaging, The Institute of Cancer Research and The Royal Marsden NHS Foundation Trust, London, SW7 3RP United Kingdom; 2 Cancer Research UK Cancer Therapeutics Unit, Division of Cancer Therapeutics, The Institute of Cancer Research, London, SW7 3RP United Kingdom; 3 Drug Development Unit, The Royal Marsden NHS Foundation Trust, London, SW7 3RP United Kingdom; 4 Division of Clinical Studies, The Institute of Cancer Research, London, SW7 3RP United Kingdom; 5 Present Address: Abbott Ireland Diagnostics Division, Pregnancy and Fertility Team, Lisnamuck, Longford, Ireland; 6 Present Address: The University of Texas MD Anderson Cancer Center, Houston, TX USA

**Keywords:** Cancer metabolism, Cancer imaging

## Abstract

**Background:**

AKT is commonly overexpressed in tumours and plays an important role in the metabolic reprogramming of cancer. We have used magnetic resonance spectroscopy (MRS) to assess whether inhibition of AKT signalling would result in metabolic changes that could potentially be used as biomarkers to monitor response to AKT inhibition.

**Methods:**

Cellular and metabolic effects of the allosteric AKT inhibitor MK-2206 were investigated in HT29 colon and PC3 prostate cancer cells and xenografts using flow cytometry, immunoblotting, immunohistology and MRS.

**Results:**

In vitro treatment with MK-2206 inhibited AKT signalling and resulted in time-dependent alterations in glucose, glutamine and phospholipid metabolism. In vivo, MK-2206 resulted in inhibition of AKT signalling and tumour growth compared with vehicle-treated controls. In vivo MRS analysis of HT29 subcutaneous xenografts showed similar metabolic changes to those seen in vitro including decreases in the tCho/water ratio, tumour bioenergetic metabolites and changes in glutamine and glutathione metabolism. Similar phosphocholine changes compared to in vitro were confirmed in the clinically relevant orthotopic PC3 model.

**Conclusion:**

This MRS study suggests that choline metabolites detected in response to AKT inhibition are time and microenvironment-dependent, and may have potential as non-invasive biomarkers for monitoring response to AKT inhibitors in selected cancer types.

## Background

The AKT/PKB (Protein Kinase B) serine/threonine kinase, with three different isoforms: AKT1, AKT2 and AKT3, is one of the core components of the PI3K signalling cascade, regulating cell proliferation, survival and metabolism, and is frequently activated in cancer.^1^ Multiple AKT inhibitors are now at various stages of clinical development.^2–4^ AKT inhibitors fall predominantly into two classes: ATP-competitive inhibitors and allosteric inhibitors of AKT.^2–4^ MK-2206 is a potent oral allosteric pan-AKT inhibitor with potential anti-neoplastic activity and is currently being evaluated in numerous clinical trials.^2–4^ Single-agent trials with this agent have generally shown anti-proliferative, rather than anti-tumour activity, with stable disease identified as the best overall response.^5–7^ Therefore, identification of non-invasive biomarkers of target inhibition and potentially of tumour response would be of value in the clinical development of the AKT inhibitor MK-2206.

Reprogrammed metabolism is one of the hallmarks of cancer.^8,9^ As many oncogenic signalling pathways that regulate cancer have also been shown to regulate metabolism,^10,11^ targeting those signalling pathways with drugs such as MK-2206 is expected to impact on metabolic intermediates. Assessment of the metabolic effects of drug treatment using functional imaging modalities, such as magnetic resonance spectroscopy (MRS) and metabolic PET, to provide an early treatment response biomarkers to molecularly targeted drugs is being increasingly investigated for clinical biomarker discovery.^12–16^

MRS provides a non-invasive and non-ionising method of detecting various tissue metabolites in vitro, ex vivo and in vivo.^17^ Numerous studies have investigated MRS-detectable metabolic biomarkers in response to novel targeted therapies that are in pre-clinical development or early phase clinical evaluation including inhibitors of HSP90, MAPK, HDAC, PI3K/AKT/mTOR and related pathways, reviewed in.^12–14^

Using MRS, we and others have previously reported alterations in the levels of choline metabolites and/or lactate in response to different PI3K/mTOR pathway inhibitors in vitro and in vivo in various cancer models.^18–28^ However to the best of our knowledge, metabolic biomarkers for AKT inhibitors have only been evaluated in vitro and ex vivo in breast cancer models,^26,27,29^ and there are no previous metabolic biomarker studies in vivo in tumour xenografts. In one study,^29^ treatment of MCF-7 and MDA-MB-231 breast cancer cells with the alkylphospholipid AKT inhibitor perifosine resulted in decreases in PC and lactate production. Two studies reported different results using the allosteric AKT inhibitor MK-2206, which provides greater specificity, reduced side-effects and less toxicity compared to alkylphospholipid AKT inhibitors.^4^ A decrease in PC levels was observed in vitro in MDA-MB-468 breast cancer cells,^27^ while an increase in PC and a decrease in lactate levels were detected ex vivo in basal-like breast cancer tumours following treatment with MK-2206.^26^

In view of the inconsistent published findings with the allosteric AKT inhibitor MK-2206, and the lack of in vivo studies in cancer models, we set out to assess the metabolic changes in response to MK-2206 both in vitro and in vivo in subcutaneous and orthotopic animal xenograft models of colon and prostate cancer, with potential to develop these metabolic changes as non-invasive biomarkers for monitoring response in clinical trials.

Our results show that treatment with the AKT inhibitor MK-2206 results in metabolic changes detectable with MRS. Importantly, a decrease in the total choline (tCho)/water ratio was observed in the more clinically relevant orthotopic model of the PC3 prostate cancer and therefore may provide a potential non-invasive biomarker for monitoring response to MK-2206 during clinical trials.

## Materials and methods

### Cell culture and treatment

The human PTEN null PC3 prostate adenocarcinoma and *PIK3CA* mutant HT29 colorectal carcinoma cell lines (American Type Culture Collection) were cultured in DMEM (Life Technologies) supplemented with 10% fetal calf serum (PAA labs Ltd), 100 U/mL penicillin, and 100 μg/mL streptomycin (Life Technologies) at 37 °C in 5% CO_2_. Cell viability was routinely > 90%, as judged by trypan blue exclusion. All cell lines were shown to be mycoplasma free using a PCR-based assay (Surrey Diagnostics Ltd) and were authenticated in our laboratory by short tandem repeat profiling.

Both cell lines were treated with the orally active, highly selective non-ATP competitive allosteric AKT inhibitor MK-2206 (Merck & Co., Inc.). GI_50_ values (concentrations causing 50% inhibition of proliferation for tumour cells) were determined using the sulforhodamine B assay following 96 h continuous exposure to compounds.^30^ At the required time points, cells underwent trypsinization and trypan blue exclusion assay.^19^ The effect of treatment on cell number was monitored by counting the number of viable attached cells in a treated flask and comparing that number with the number of attached cells in a control flask.

### Flow cytometry

Cell cycle analysis was performed as previously described.^19^

### Immunoblotting

Western blotting was performed as previously described.^19^ Western blots were probed for pAKT (Ser473; 4060), AKT (9272), pRPS6 (Ser240/244; 2215), RPS6 (2217), HK2 (2106), PARP (9542), LDHA (3582), β-Actin (4967), all from Cell Signaling Technology, and CHKA (HPA0241153) from Sigma. Blots were revealed with peroxidase-conjugated secondary anti-rabbit (GE Healthcare NA9340) or anti-mouse (DAKO P0260) antibodies followed by ECL chemiluminescence solution (Amersham Biosciences).

### In vitro ^1^H and ^31^P-MRS of cell extracts

The same number of cells per flask were seeded at the beginning of the experiment then at the selected time points; cells were pooled from the number of flasks required to achieve an average cell number of 3 × 10^7^ cells, which differed depending on the expected effect of treatments on cell number. To obtain an MR spectrum, cells were extracted from cultured cells using the dual phase extraction method, as previously described.^19,31^ Briefly, cells were rinsed with ice-cold saline and fixed with 10 mL of ice-cold methanol. Cells were then scraped off the surface of the culture flask and collected into tubes. Ice-cold chloroform (10 mL) was then added to each tube followed by an equal volume of ice-cold deionised water. Following phase separation, the solvent in the upper methanol/water phase was removed by lyophilisation. Prior to acquisition of the MRS spectra, the water-soluble metabolites were resuspended in deuterium oxide (D_2_O) for ^1^H-MRS or D_2_O with 10 mM EDTA (pH 8.2) for ^31^P-MRS. For extracellular metabolite analysis, 500 μL of cell growth medium was mixed with 100 μL of D_2_O containing sodium 3-trimethylsilyl-2,2,3,3-tetradeuteropropionate as an internal reference (TSP; 2.7 mM). ^1^H-MRS and ^1^H-decoupled ^31^P-MRS spectra were acquired at 25 °C on a 500 MHz Bruker spectrometer (Bruker Biospin, Coventry, UK) using a 90° flip angle, a 1 s relaxation delay, spectral width of 12 ppm, 64 K data points, and HDO resonance suppression by presaturation for ^1^H-MRS and a 30° flip angle, a 1 s relaxation delay, spectral width of 100 ppm, and 32 K data points for ^31^P. Metabolite contents were determined by integration and normalised relative to the peak integral of an internal reference [TSP (4.8 mM) for ^1^H-MRS, and methylenediphosphonic acid (MDPA; 2 mM) for ^31^P-MRS] and corrected for signal intensity saturation (^31^P-MRS) and the number of cells extracted per sample.

### In vivo tumour propagation

All animal experiments were performed in accordance with local and national ethical review panel, the UK Home Office Animals (Scientific Procedures) Act 1986 and the United Kingdom Coordinating Committee on Cancer Research Guidelines for the Welfare of Animals in Experimental Neoplasia.^32^

### Subcutaneous HT29 and PC3 tumour xenografts

Male NCr nude mice were injected subcutaneously in the flank with 5 × 10^6^ HT29 (human colon) or PC3 (human prostate) carcinoma cells. Tumour volume was calculated by measuring the length, width, and depth using calipers and the ellipsoid formula L x W x D x (π/6). Once the tumours reached ~400 mm^3^, the animals were divided to two groups. One group was treated with 2 doses of 120 mg/kg of MK-2206 on alternate days (Day 1 and 3) via p.o. and the other group with vehicle alone (10% DMSO in saline).

### Orthotopic PC3 tumour xenografts

PC3 cells (5 × 10^5^) were inoculated in the ventral prostate gland of nude mice. Once the tumours were palpable, animals were treated with 2 doses of 120 mg/kg of MK-2206 on alternate days (Day 1 and 3) via p.o. or vehicle alone (10% DMSO in saline).

### In vivo MRS of HT29 and PC3 tumours

Mice were anesthetised with a single intraperitoneal injection of a fentanyl citrate (0.315 mg/mL) plus fluanisone [10 mg/mL (Hypnorm; Janssen Pharmaceutical Ltd., High Wycombe, UK)], midazolam [5 mg/mL (Hypnovel; Roche, Welwyn Garden City, UK)], and sterile water (1:1:2) at a dose of 9 mL/kg. They were placed in the bore of a 7 Tesla Bruker MR System spectrometer (Bruker Biospin, Coventry, United Kingdom) with HT29 and PC3 tumours positioned in the centre of a 15 mm two-turn ^1^H/^31^P surface coil. In vivo localised PRESS ^1^H-MRS of the tumours was carried out at 37 °C on Day 0 (before treatment) and the last day of treatment (Day 3). 4 × 4 × 4 mm voxels were selected from fast spin-echo images and shimmed using a localised sequence. The localised PRESS with water suppression was used to detect choline with a repetition time of 4 s, echo times 136 ms and 64 transients. 4 transients were used to acquire the unsuppressed water spectra with the same acquisition parameters as above. Image-selected in vivo spectroscopy (ISIS) ^31^P-MR spectra were also obtained in subcutaneous PC3 tumours with a repetition time of 2 s and 64 transients. After the final MRS scan, tumours were excised and stored at for subsequent ex vivo ^1^H and ^31^P-MRS, MSD^®^ assays or immunohistochemical analysis.

^1^H and ^31^P-MR spectra were analysed using the JMRUI programme to pre-process, fit and quantify peak areas of the observed metabolites. Choline levels are expressed as a ratio relative to the water (tCho/water) signal following corrections for the number of averages and receiver gains, as these two parameters were different for the acquisitions of water and choline spectra. Phosphomonoesters (PMEs) were expressed as ratios relative to total phosphorus (PMEs/total P) signals.

### Meso scale discovery (MSD^®^) assay

Tumour pharmacodynamic biomarkers for MK-2206 were assessed by a MSD^®^ multispot electrochemiluminescence immunoassay system to detect pP70S6K (Thr421/Ser424), total P70S6K, pAKT (Ser473), pAKT (Thr308), total AKT, pRPS6 (Ser235/236), pRPS6 (Ser240/244) and total RPS6 in 10 mg tumour lysate of vehicle and MK-2206 treated tumours according to the manufacturer’s instructions (Meso Scale Discovery, Gaithersburg, USA).

### Ex vivo MRS of tumour extracts

100–200 mg of the freeze-clamped tumours were finely grinded in liquid nitrogen and extracted using ice-cold methanol, water and chloroform (1:1:1). The aqueous phase was separated, freeze-dried and reconstituted in 650 µl D_2_O. 50 µL of 44 mM TSP in D_2_O was added to the samples for ^1^H chemical shift calibration and quantification. The samples were then placed in 5 mm NMR tubes and sample pH was adjusted to 7 using perchloric acid or potassium hydroxide. ^1^H-MRS of the tumour extracts was performed on a Bruker 500 MHz nuclear magnetic resonance system (Bruker Biospin, Coventry, United Kingdom) and spectra were acquired using a pulse and collect NMR sequence with presaturation for water suppression; 7500 Hz spectral width, 32 K time domain points, 2.7 s relaxation delay and 256 scans at 298 K. After ^1^H-MRS, 50 μL of 60 mM EDTA was added to each sample for chelation of metal ions and 25 μL of 10 mM MDPA was added to the samples for ^31^P chemical shift calibration and quantitation. The pH was again adjusted to 7 and ^31^P-MRS was performed with 12,000 Hz spectral width, 32 K time domain points, 5 s relaxation delay and 3000 scans at 298 K.^33^

MR spectra were analysed using the Bruker Topspin-3.2 software package (Bruker Biospin, Coventry, UK). Spectra were processed by using exponential multiplication with a line broadening of 0.3 Hz and 3 Hz for ^1^H and ^31^P-MR spectra, respectively, then followed by Fourier transform, zero- and first-order phase correction, baseline correction and spectral peak integration integration. Spectral assignments were based on literature values.^33,34^ Water-soluble metabolites measured by ^1^H and ^31^P-MRS were quantified relative to TSP or MDPA, respectively, and standardised to tumour weight.^33^

### Immunohistochemistry

Tumour xenografts were fixed in 10% formaldehyde and routinely processed for paraffin embedding. For histological evaluation, 5 μm-thick paraffin wax sections were cut and stained with haematoxylin and eosin. Expression of caspase-3 (apoptotic marker), CD31 (micro-vessel density) and Ki67 (proliferation marker) were determined by immunohistochemistry, using the streptavidin-biotin peroxidase technique. Briefly, sections of 5 μm were deparaffinised in xylene and rehydrated through graded ethanol concentrations up to distilled water for 30 min. Antigen retrieval was performed by microwaving the sections in 10 mM sodium citrate buffer pH 6 at 10 min intervals for a total of 20 min and cooling for 1 h at room temperature (RT). Endogenous peroxidase activity was blocked by incubating the sections in a solution of 3% hydrogen peroxide for 20 min at RT. After washing in PBS (phosphate buffer saline), sections were incubated with the primary polyclonal rabbit anti-human caspase-3 (1:50, Abcam ab2302), monoclonal rabbit anti-human CD31 (1:50, Millipore 04–1074) mouse monoclonal anti-human Ki67 (1:75, DAKO M7240) antibodies, overnight at 4 °C. The sections were washed with PBS and incubated with a biotinylated secondary antibody for 45 min, followed by an incubation with streptavidin-biotin horseradish peroxidase complex (DAKO) for another 45 min, at RT. Staining was carried out using a solution 3,3′-diaminobenzidine (DAB-Sigma), and lightly counterstained with Harris haematoxylin.

### Evaluation of staining

Sections known to express high levels of caspase-3 (pancreas), CD31 (liver) and Ki67 (tonsil) were included as positive controls, while negative control slides were incubated with PBS. Caspase-3 and Ki67 immuno-stained slides were assessed by light microscopy and scored with ImageJ (1.50i). A semi-quantitative method was used to score the microvessels stained with CD31.^35^ Three fields showing the highest number of microvessels were selected using light microscopy and the number of micovessels in these fields were then manually counted and averaged. Each section was scored by 2 independent observers at 200× magnification.

### Statistical analysis

Data are presented as the mean ± SD (in vitro) or mean ± SEM (in vivo and ex vivo) and *n* ≥ 3. Statistical significance of differences was determined by Student’s standard *t*-tests with a *P* value of ≤0.05 considered to be statistically significant.

## Results

### In vitro investigation of molecular and metabolic effects of treatment with MK-2206 in PC3 human prostate cancer cells

**Fig. 1:**
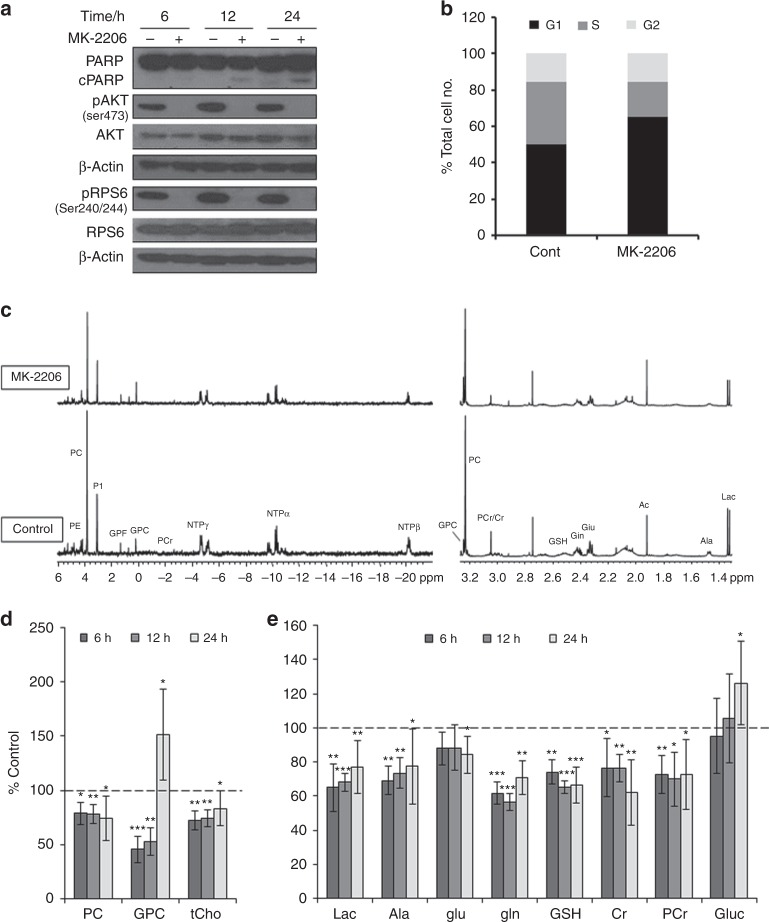
Molecular and metabolic changes caused by treatment with MK-2206 in PC3 prostate cancer cells. **a** Representative immunoblots showing changes in molecular markers demonstrating AKT inhibition and induction of apoptosis as evidenced by cleaved PARP. β-Actin is used as a loading control. **b** Flow cytometry analysis histograms showing cell cycle distribution of cells with vehicle treatment (DMSO, control), or following treatment with MK-2206 (5xGI_50_) at 24 h post treatment, *P* *<* 0.002 for G1&S phases. **c** Representative in vitro ^31^P-MR spectra (left) and expansion of ^1^H-MR spectra region (1.3–3.3 ppm; right) showing choline–containing metabolites, Cr/PCr, lactate (Lac) and amino acids (Ala = alanine; Glu = glutamate; Gln = glutamine; GSH = glutathione). A summary of ^1^H-MRS metabolic changes caused by MK-2206 treatment (5xGI_50_, 24 h) of PC3 prostate cancer cells: **d** Choline-containing metabolites. **e** Amino acids, Cr/PCr and glycolytic intermediates. Results are expressed as %T/C and presented as mean ± SD, *n* ≥ 5. Statistically significant differences from the control ^*^P ≤ 0.05; ^**^P ≤ 0.01; ^***^P ≤ 0.001

The PTEN null human prostate cell line PC3 was treated with MK-2206 for 6, 12 and 24 h at a pharmacologically active concentration corresponding to 5xGI_50_ (GI_50_ = 5 μM). Inhibition of the AKT pathway was evident at all time points as indicated by decreased phosphorylation of AKT (Ser473) and RPS6 (Ser240/244) in treated cells compared to their controls (Fig. 1a). Treatment with MK-2206 also induced apoptosis which was evident at 12 and 24 h following treatment as indicated by PARP cleavage detected by immunoblotting (Fig. 1a). Inhibition of cell growth (down to 66 ± 10%, *P* *=* 0.0001) and a G1 cell cycle arrest was only detectable at 24 h post treatment (Fig. 1b).

^31^P-and ^1^H-MRS of aqueous extracts from PC3 cells treated in vitro with the AKT inhibitor MK-2206 (5xGI_50_) was used to identify potential biomarkers of AKT pathway inhibition compared to controls (Fig. 1c). Analysis of metabolites detected with ^31^P-MRS showed a significant decrease (*P* *≤* 0.02) in the levels of phosphoethanolamine (PE), phosphocholine (PC) and NTP which was evident at 6 h and was maintained at 24 h (Table 1). Levels of glycerophosphoethanolamine (GPE) and glycerophosphocholine (GPC) were reduced for up to 12 h (*P* *≤* 0.001) but then a significant increase (*P* *≤* 0.01) was observed at 24 h (Table 1). A significant decrease (*P* *≤* 0.04) in the levels of phosphocreatine (PCr) was also detected at 12 h and was maintained at 24 h following treatment with MK-2206. ^1^H-MRS confirmed changes in PC and GPC detected with ^31^P-MRS together resulted in a significant decrease (*P* *≤* 0.04) in tCho levels (Fig. 1d). Furthermore, significant decreases (*P* *≤* 0.05) in lactate, alanine, glutamine, glutathione, creatine (Cr) and PCr levels were detected over the time course of treatment (Fig. 1e). A significant (*P* *≤* 0.05) decrease in glutamate and increase in glucose were also found following 24 h of MK-2206 treatment (Fig. 1e). We also assessed the metabolic effects of MK-2206 at a lower concentration equivalent to 3xGI_50_ for 24 h. This resulted in inhibition of AKT signalling and cellular growth (down to 84 ± 8%, *P* *=* 0.008) as well as a G1 cell cycle arrest compared to controls, but did not induce apoptosis as detemined by cleaved PARP (Supplementary Figure S1A). ^31^P-MRS showed similar changes in PC, PE, PCr and NTP to those observed with MK-2206 at 5xGI_50_, but levels of GPE and GPC were not affected (Table 1). Similarly, decreases in PC, tCho, lactate, alanine, glutathione, Cr and PCr were detected using ^1^H-MRS, while glutamate, glutamine and glucose levels remained unchanged relative to controls (Supplementary Figure S1B and C).

**Table 1:** Analysis of ^31^P-MRS-detected metabolic changes following inhibition with MK-2206 in

	PC3							
	6 h (5xGI_50_)	*P*	12 h (5xGI_50_)	*P*	24 h (5xGI_50_)	*P*	24 h (3xGI_50_)	*P*
PE	62 ± 16	0.008	47 ± 11	0.001	23 ± 16	0.0001	43 ± 26	0.01
PC	74 ± 14	0.02	78 ± 11	0.02	69 ± 13	0.001	71 ± 5	0.0004
GPE	66 ± 34	ns	50 ± 12	0.001	320 ± 104	0.005	153 ± 40	ns
GPC	32 ± 16	0.001	36 ± 11	0.0003	225 ± 63	0.007	121 ± 27	ns
PCr	74 ± 28	ns	54 ± 31	0.04	38 ± 24	0.007	46 ± 24	0.01
NTP	63 ± 17	0.01	56 ± 12	0.002	68 ± 18	0.005	82 ± 11	0.03

	HT29	
	24 h (5xGI_50_)	*P*
PE	60 ± 9	0.001
PC	67 ± 6	0.0001
GPE	132 ± 26	0.04
GPC	169 ± 31	0.004
PCr	59 ± 8	0.0001
NTP	89 ± 7	0.02

Data are expressed as %T/C and presented as the mean ± SD, *n* ≥ 4

Two-tailed unpaired *t* test was used to compare results in treated cells to controls within the same time-point

### In vitro investigation of molecular and metabolic effects of treatment with MK-2206 in HT29 human colon cancer cells

To test for the generality of the MRS-detected data, we also treated *PIK3CA* mutant HT29 colorectal carcinoma cells with MK-2206 at 5xGI_50_ (GI_50_ = 0.4 μM) for 24 h. Similar to PC3 prostate cells, treatment with MK-2206 resulted in inhibition of AKT signalling and a G1 cell cycle arrest but no effects on cell number nor apoptosis were detected relative to controls (Supplementary Figure S2A and B). Representative ^31^P and ^1^H-MR spectra are shown in Supplementary Figure S2C. As in PC3 cells, ^31^P-MRS analysis showed significant decreases (*P* *≤* 0.04) in PE, PC, PCr and NTP and increases in GPE and GPC in spectra from MK-2206 treated cells compared to their controls (Table 1). ^1^H-MRS confirmed changes in PC, GPC and further showed a reduction in tCho (Supplementary Figure S2D). Consistent with PC3 cells, significant decreases (*P* *≤* 0.04) in lactate, alanine, glutamate, glutamine, glutathione, Cr and PCr were also observed in HT29 cells following MK-2206 treatment (Supplementary Figure S2E). In contrast to PC3 cells, treatment with MK-2206 reduced glucose levels in HT29 cells (*P* *<* 0.02; Supplementary Figure S2E).

### Assessment of mechanisms underlying the in vitro MRS detected metabolic changes following treatment with MK-2206

We have used immunoblotting to identify the effects of AKT inhibition with MK-2206 on enzymes involved in choline and glucose metabolism. A decrease in choline kinase alpha (CHKA) expression levels compared to control cells was observed over the time course of treatment with MK-2206 in PC3 and following 24 h treatment with MK-2206 in HT29 cells (Supplementary Figure S3A and B). For the glycolytic metabolic changes, reductions in the protein expression levels of the glycolytic enzymes including hexokinase II (HK2) and lactate dehydrogenase alpha (LDHA) were detected in both cell lines following treatment with MK-2206 (Supplementary Figure S3A and B).

Next, in order to determine whether the changes in intracellular metabolites could be due to alterations in metabolic flux, we used ^1^H-MRS to measure levels of metabolites in the growth media of control and treated cells. In the PC3 cells (Supplementary Figure S4A), treatment with MK-2206 (5xGI_50_) caused no significant changes in external metabolites at 6 h compared to controls. However, significant increases (*P* < 0.05) in the levels of alanine, glutamine and choline were observed at 12 h following treatment. High levels of all metabolites were detected in growth media of 24 h treated cells compared to controls resulting from the release of metabolites from fragmented apoptotic cells. In contrast, 24 h treatment with MK-2206 at 3xGI_50_ only caused a significant increase (*P* = 0.01) in the level of choline compared to controls. Increases in the levels of metabolites present in the growth media from HT29 cells treated with MK-2206 (5xGI_50_) were detected but did not reach significance relative to controls (Supplementary Figure S4B).

### In vivo investigation of molecular and metabolic effects of treatment with MK-2206 in subcutaneous HT29 colon xenografts

**Fig. 2:**
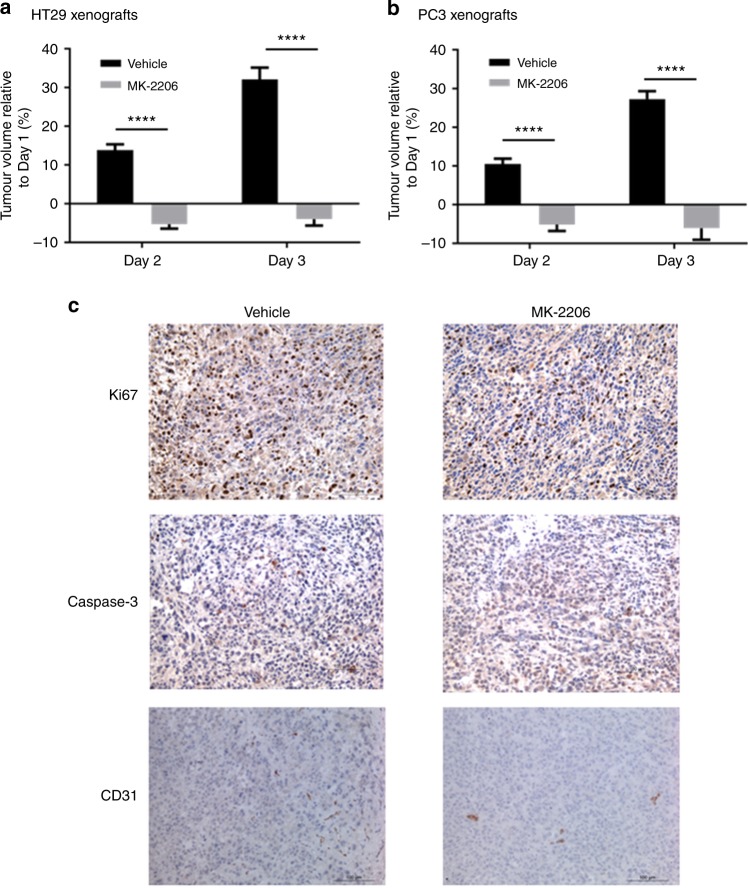
Tumour volume and histological changes in subcutaneous tumours following MK-2206 treatment. Percentage change in HT29 (**a**) and subcutaneous PC3 (**b**) tumour volumes (relative to Day 1) following 2 doses (Day 1 and 3) of 120 mg/kg of MK-2206 on alternate days via p.o. (*n* = 10) or vehicle alone (10% DMSO in saline), minimum *n* = 10. Data are expressed as mean ± SEM, ****P *<* 0.0001. **c** Immunohistochemistry of Ki67, caspase-3 and CD31 expressions (brown staining) in vehicle-treated control (left column) and MK-2206 treated (right column) subcutaneous PC3 xenografts (right column). Magnification 200×

Significant tumour growth inhibition was observed in HT29 xenografts after 2 doses (Day 1 and 3) of MK-2206 (120 mg/kg per dose) when compared with vehicle-treated controls (Fig. 2a). AKT inhibition was confirmed by reductions in the phosphorylation of P70S6K, RPS6 (Ser235/236), AKT (Ser473) and AKT (Thr308; Supplementary Figure S5). In vivo ^1^H-MRS showed a significant decrease (*P* = 0.04) in the ratio of tCho/water signal in HT29 xenografts after MK-2206 treatment (Table 2). The in vivo change in tCho/water was confirmed by lower PC, GPC and GPE levels in ex vivo ^31^P-MRS analysis of MK-2206 treated tumour extracts when compared with vehicle controls (Table 2). Lower levels of glutamine, glutamate, aspartate, glycine, glutathione and Cr were also seen in MK-2206 treated tumours when compared with controls (Table 2). Phosphocreatine, ATP + ADP, NTP [0.68 ± 0.07 (control) versus 0.48 ± 0.03 (MK-2206) µmol/g wet weight; *P* = 0.015] and NDP [0.49 ± 0.02 (control) versus 0.30 ± 0.02 (MK-2206) µmol/g wet weight; *P* = 0.0004] levels were also found to reduce in the MK-2206 treated group (Table 2). No change in glucose and lactate levels, microvessel density, necrosis, proliferation or apoptosis was found in MK-2206 treated HT29 tumours when compared to vehicle controls using immunohistochemistry.

**Table 2:** In vivo and ex vivo ^1^H and ^31^P-MRS metabolic analysis of HT29 subcutaneous tumours and extracts following MK-2206 treatment

In vivo ^1^H-MRS of subcutaneous HT29 xenografts				
	Vehicle-control (*n* = 5)		MK-2206 (*n* = 5)	
	Pre	Post	Pre	Post
Corrected tCho/water ratio x 10^−3^	8.05 ± 1.11	6.25 ± 1.84	9.22 ± 1.42	4.33 ± 1.03
	*P* *=* 0.31		**P *=* 0.04	

Ex vivo ^1^H and ^31^P-MRS of subcutaneous HT29 tumour extracts			
	Vehicle-control	MK-2206	*P*
PE	1.27 ± 0.10	1.28 ± 0.11	0.96
PC	1.95 ± 0.12	1.60 ± 0.09	0.04*
GPE	1.21 ± 0.14	0.87 ± 0.07	0.03*
GPC	2.51 ± 0.13	1.94 ± 0.18	0.04*
Lactate	5.81 ± 0.58	7.78 ± 0.92	0.10
Alanine	1.49 ± 0.15	1.27 ± 0.08	0.25
Glucose	0.79 ± 0.10	0.70 ± 0.13	0.61
Glutamine	1.01 ± 0.06	0.72 ± 0.02	0.002*
Glutamate	2.67 ± 0.23	1.56 ± 0.30	0.02*
Aspartate	0.35 ± 0.04	0.20 ± 0.05	0.05*
Glycine	0.67 ± 0.11	0.37 ± 0.06	0.04*
Glutathione	1.30 ± 0.11	0.85 ± 0.08	0.007*
Creatine	3.82 ± 0.21	2.87 ± 0.19	0.008*
Phosphocreatine	1.20 ± 0.08	0.74 ± 0.08	0.004*
ATP + ADP	1.38 ± 0.08	1.07 ± 0.07	0.02*

Data are expressed as µmol/g wet weight and presented as the mean  ±  sem, *n*  ≥  5 in each group. Two-tailed unpaired *t* test was used to compare MK2206-treated tumour extracts with vehicle-treated controls and ^*^P  ≤  0.05 is considered significant.

**Statistically significant when compared the pre-MK-2206 treatment values with post-treatment. Two-tailed paired *t* test was used and data are expressed as mean ± sem

### In vivo investigation of molecular and metabolic effects of treatment with MK-2206 in subcutaneous PC3 prostate tumour xenografts

Similar to MK-2206 treated HT29 xenografts, significant tumour growth inhibition was also observed in PC3 xenografts after 2 doses (Day 1 and 3) of MK-2206 (120 mg/kg per dose) when compared with vehicle-treated controls (Fig. 2b) and AKT inhibition was confirmed by the reductions in the phosphorylation of P70S6K, RPS6 (Ser235/236), AKT (Ser473) and AKT (Thr308) (Supplementary Figure S6). In vivo ^1^H-MRS did not show a change in the ratio of tCho/water signal in either control or MK-2206 treated PC3 xenografts (Table 3). However, a significant increase (*P* = 0.02) in the ratio of PMEs/total *P* signal was found in MK-2206 treated PC3 xenografts by in vivo ^31^P-MRS (Table 3), with this in vivo change attributable to a significant increase (*P* = 0.03) in PE measured by ^31^P-MRS of MK-2206 treated PC3 tumour extracts (Table 3). Significant decreases (*P* ≤ 0.04) in GPC, GPE and lactate and increase in glutamine were also found in MK-2206 treated PC3 tumour extracts when compared with vehicle controls (Table 3). No change in tumour bioenergetics was observed in this tumour model following MK-2206 treatment. Immunohistochemical analysis (Fig. 2c) on the tumour samples showed significantly decreased microvessel density (CD31) in MK-2206 treated tumours (9 ± 2 stained blood vessels average over 3 fields) when compared to vehicle-controls (14 ± 2; *P* *=* 0.05). Using immunohistochemistry, no change in necrosis, proliferation or apoptosis was found in MK-2206 treated tumours when compared to controls (Fig. 2c).

**Table 3:** In vivo and ex vivo ^1^H and ^31^P-MRS metabolic analysis of subcutaneous PC3 tumours and extracts following MK-2206 treatment

In vivo ^1^H and ^31^P-MRS of subcutaneous PC3 tumours				
	Vehicle-control		MK-2206	
	Pre	Post	Pre	Post
Corrected tCho/water ratio x 10^−3^ (*n* = 4 in each group)	3.63 ± 0.38	3.80 ± 0.64	3.26 ± 0.26	3.61 ± 0.17
	*P* *=* 0.66		*P* = 0.31	
PME/total P ratio (*n* = 5 in each group)	0.11 ± 0.01	0.12 ± 0.01	0.14 ± 0.01	0.17 ± 0.02
	*P* = 0.50		**P = 0.02	

Ex vivo ^1^H and ^31^P-MRS of subcutaneous PC3 tumour extracts			
	Vehicle-control	MK-2206	*P*
PE	1.03 ± 0.10	1.45 ± 0.13	0.03*
PC	1.53 ± 0.05	1.60 ± 0.20	0.75
GPE	0.44 ± 0.03	0.28 ± 0.04	0.02*
GPC	1.54 ± 0.14	0.94 ± 0.19	0.04*
Lactate	9.34 ± 0.85	7.14 ± 0.44	0.04*
Alanine	1.39 ± 0.13	1.49 ± 0.23	0.74
Glucose	0.45 ± 0.06	0.41 ± 0.03	0.48
Glutamine	1.20 ± 0.16	2.74 ± 0.48	0.02*
Glutamate	3.64 ± 0.41	3.02 ± 0.37	0.28
Glycine	1.21 ± 0.18	1.39 ± 0.16	0.48
Glutathione	1.53 ± 0.15	1.46 ± 0.12	0.74
Creatine	1.08 ± 0.09	1.38 ± 0.24	0.32
Phosphocreatine	0.39 ± 0.05	0.53 ± 0.05	0.08
ATP + ADP	0.84 ± 0.05	0.89 ± 0.07	0.63

Data are expressed as µmol/g wet weight and presented as the mean  ±  sem, *n*  ≥  5 in each group. Two-tailed unpaired *t* test was used to compare MK2206-treated tumour extracts with vehicle-treated controls and ^*^P  ≤  0.05 is considered significant. Aspartate was not detected.

**Statistically significant when compared the pre-MK-2206 treatment values with post-treatment. Two-tailed paired *t* test was used and data are expressed as mean ± sem

PME phosphomonoesters, total P total phorphorus signal

### In vivo investigation of molecular and metabolic effects of treatment with MK-2206 in orthotopic PC3 prostate xenografts

Next we wanted to examine the metabolic response to MK-2206 in a more clinically relevant in vivo model. Orthotopic PC3 tumours were propagated, treated with MK-2206 (2 doses of 120 mg/kg on Day 1 and 3) and studied by ^1^H-MRS. AKT inhibition was confirmed by the reductions in phosphorylated RPS6 (Ser240/244), AKT (Ser473) and AKT (Thr308; Supplementary Figure S7). In vivo ^1^H-MRS showed that the tCho/water ratio was significantly reduced (*P* = 0.02) in orthotopic PC3 tumours after MK-2206 treatment, with this reduction attributable to a significant decrease (*P* = 0.003) in PC as measured ex vivo by ^31^P-MRS analysis of the tumour extracts (Table 4). Significant decreases (*P* ≤ 0.03) in alanine and increases in glucose were also found in MK-2206 treated tumours (Table 4). No changes in tumour bioenergetics, glutamine or glutathione metabolism were observed in this tumour model following MK-2206 treatment. No change in microvessel density, necrosis, proliferation or apoptosis was found in MK-2206 treated tumours when compared to vehicle-controls.

**Table 4:** In vivo and ex vivo ^1^H and ^31^P-MRS metabolic analysis of orthotopic PC3 tumours and extracts following MK-2206 treatment

In vivo ^1^H-MRS of orthotopic PC3 tumours				
	Vehicle-control (*n* = 3)		MK-2206 (*n* = 5)	
	Pre	Post	Pre	Post
Corrected tCho/water ratio x 10^−3^	5.11 ± 0.68	4.25 ± 0.47	5.03 ± 0.69	3.92 ± 0.56
	*P* *=* 0.36		**P = 0.02	

Ex vivo ^1^H and ^31^P-MRS of orthotopic PC3 tumour extracts			
	Vehicle-control	MK-2206	*P*
PE	1.58 ± 0.18	1.20 ± 0.13	0.14
PC	1.80 ± 0.06	1.47 ± 0.06	0.003*
GPE	0.46 ± 0.03	0.45 ± 0.05	0.95
GPC	1.79 ± 0.35	2.16 ± 0.17	0.40
Lactate	5.32 ± 0.65	3.96 ± 0.50	0.14
Alanine	1.09 ± 0.09	0.78 ± 0.07	0.03*
Glucose	0.33 ± 0.04	0.79 ± 0.15	0.009*
Glutamine	1.28 ± 0.16	0.97 ± 0.13	0.18
Glutamate	3.10 ± 0.25	2.50 ± 0.16	0.08
Aspartate	0.10 ± 0.02	0.08 ± 0.01	0.39
Glycine	1.06 ± 0.05	1.01 ± 0.14	0.76
Glutathione	1.59 ± 0.32	1.07 ± 0.09	0.19
Creatine	1.30 ± 0.16	1.17 ± 0.53	0.26
Phosphocreatine	0.36 ± 0.10	0.28 ± 0.04	0.50
ATP + ADP	0.98 ± 0.08	0.89 ± 0.08	0.46

Data are expressed as µmol/g wet weight and presented as the mean  ±  sem, *n*  ≥  5 in each group. Two-tailed unpaired *t* test was used to compare MK2206-treated tumour extracts with vehicle-treated controls and ^*^P  ≤  0.05 is considered significant.

**Statistically significant when compared the pre-MK-2206 treatment values with post-treatment. Two-tailed paired *t* test was used and data are expressed as mean ± sem

## Discussion

AKT is a central component of the PI3K signalling pathway, influencing multiple processes that are directly involved in tumourigenesis. Targeting AKT is therefore a highly attractive anti-cancer strategy and several AKT inhibitors are currently in different phases of clinical trials.^2–4^ As with most cancer targeted therapy, AKT inhibitors were shown to cause anti-proliferative, rather than anti-tumour activity, with stable disease identified as the best overall response.^5–7^ Therefore, the use of conventional, anatomically based end-points such as RECIST is inadequate.^15,36^ AKT also plays a pivotal role in the metabolic reprogramming of cancer, providing a rationale for the use of non-invasive functional imaging techniques (such as MRS or PET) as alternative methods to monitor response to this targeted therapy.^12–16^

We used MRS both in vitro and in vivo to identify whether inhibition of AKT signalling using the allosteric pan-AKT inhibitor MK-2206 would result in metabolic changes that can potentially be used to monitor response to AKT inhibition in clinical trials. We performed our investigation using the human *PIK3CA* mutant colorectal carcinoma HT29 and PTEN null prostate carcinoma PC3 cancer models as AKT signalling is involved in the tumourigenesis of colorectal and prostate cancers and AKT inhibitors are in clinical evaluation for both cancer types.^37–39^

MK-2206 consistently resulted in the reduction of AKT and its downstream, mTOR, signalling pathways in PC3 and HT29 cells and tumours confirming the mechanism of action.

Treatment of PC3 cells with MK-2206 resulted in decreases in PE, PC, tCho, lactate, alanine, glutamine, glutathione, Cr, PCr and NTP levels from 6 h post-treatment onwards which was associated with AKT/mTOR pathway inhibition, but was much earlier than the G1 arrest, induction of apoptosis and the decrease in proliferation which were only evident at 24 h following treatment with MK-2206. This indicates that our detected metabolic changes are related to the inhibition of AKT/mTOR signalling rather than to the anti-proliferative effects of the treatment. In support of previous reports by ourselves and others using PI3K/mTOR/AKT inhibitors,^18,19,21,28,29^ the decrease in PC levels following MK-2206 treatment was associated with a decrease in the protein levels of CHKA, the enzyme responsible for choline phosphorylation to form PC. A decrease in the protein expression levels of the glycolytic enzymes HK2 and LDHA were also observed following AKT inhibition, suggesting mechanisms for the depletion of lactate. Higher levels of choline were also found in the tissue culture media of cells treated with MK-2206 compared to controls indicating inhibition of uptake as another mechanism for the decrease in intracellular levels of PC. Furthermore, decreased intracellular and increased extracellular levels of alanine indicate the conversion of pyruvate into alanine instead of lactate as a result of inhibition of LDHA and increased eflux of alanine following treatment with MK-2206. A decrease in the intracellular and increase in the extracellular level of glutamine was also detected in treated cells which maybe related to decreased uptake of glutamine into the cells following MK-2206 treatment.

Treatment with MK-2206 also reduced levels of GPE and GPC for up to 12 h but then an increase was observed at 24 h. The later increase in GPE and GPC might be linked to the apoptotic effects of MK-2206 observed at this time point which would lead to membrane breakdown and remodeling.^40,41^ We have previously observed an increase in GPC following treatment with some PI3K inhibitors but that was cell line dependent and, moreover, was seen only after longer inhibition periods (≥16 h) and when higher concentrations (5xGI_50_) of PI3K pathway inhibitors were used.^18–20^ This was further supported by our findings that when we used MK-2206 at a concentration equivalent to 3xGI_50_. This concentration did not cause apoptosis and had no effects on GPC or GPE levels.

Similar to PC3 cells, metabolic changes including reductions in PE, PC, tCho, lactate, alanine, glutamate, glutamine, glutathione, Cr and PCr as well as an increase in GPE and GPC were detected following treatment of HT29 colorectal carcinoma cells with MK-2206 at 5xGI_50_ for 24 h. This was associated with inhibition of AKT/mTOR signalling and a G1 cell cycle arrest. The observed changes in phospholipid and glucose metabolism are congruent with the previous reports examining the effect of the AKT inhibitors perifosine and MK-2206 on breast cancer cells,^27,29^ and suggest that choline-containing metabolites and lactate may serve as non-invasive metabolic biomarkers for monitoring the effects of AKT inhibitors.

Similar phospholipid and glutamine changes to those detected in HT29 cells were also observed in HT29 xenografts following treatment with MK-2206. These were associated with a significant tumour growth delay and pathway inhibition when compared with vehicle-treated controls. In vivo ^1^H-MRS analysis of the HT29 tumour xenografts showed a significant decrease in the ratio of tCho/water signal. This was further confirmed by significantly lower PC, GPC and GPE levels by ex vivo ^31^P-MRS of MK-2206 treated tumour extracts when compared with vehicle controls, supporting the in vitro findings and suggesting that membrane turnover is reduced following MK-2206 treatment. Tumour bioenergetics was also compromised by treatment with MK-2206 as indicated by the decrease in the levels of PCr, ATP + ADP, NTP, and NDP. Consistent with our in vitro cell data, alterations in glutamine and glutathione metabolism with decreased glutamine, glutamate, aspartate, glutathione, glycine and Cr were also found in HT29 tumours following AKT inhibition with MK-2206. No change in glucose metabolism was observed in MK-2206 treated HT29 xenografts.

Glutamine is one of the key substrates utilised by cancer cells and its metabolism is important to tumour growth, malignancy, and survival under stress.^42^ Glutamine is involved in nucleotide synthesis,^43^ and generation of the anti-oxidant glutathione.^44^ The decreases in bioenergetic metabolites, such as nucleotides and PCr following MK-2206 treatment are consistent with the observed decreases in glycine, glutamine and its downstream metabolites, such as glutamate, aspartate and Cr, suggesting that lower tumour bioenergetics following treatment maybe a consequence of changes in glutamine metabolism.

Our data also indicate that glutathione biosynthesis may be altered following MK-2206 treatment, as the total glutathione level together with its precursors, glutamine and glycine, were lower in the MK-2206 treated PC3 and HT29 cells and tumours. This is consistent with previous reports that glutathione levels are reduced in MK-2206 treated lung cancer cells^45^ and that the PI3K/AKT signalling pathway in *PIK3CA* mutant and *PTEN* mutant breast cancer cells stimulates glutathione biosynthesis, in order to counteract the effect of oxidative stress.^46^

Different changes in PC levels have been previously reported following treatment with MK-2206 in MDA-MB-468 breast cancer cells^27^ compared to basal like breast cancer tumours.^26^ This was the case with MK-2206 treated subcutaneous PC3 xenografts, where in contrast to PC3 cells, an increase rather than a decrease in the ratio of PMEs/total P signal was observed by in vivo ^31^P-MRS, and no significant difference in the tCho/water ratio was detected by in vivo ^1^H-MRS pre vs. post MK-2206 treatment. Further investigations using ex vivo MRS showed an increase in PE and a decrease in GPC and GPE in MK-2206 treated subcutaneous PC3 tumour extracts when compared with vehicle controls. These changes in choline and ethanolamine metabolites could explain the lack of change in the in vivo MRS detected tCho/water signal as it consists of PC, PE, GPC and GPE, and the increase in PMEs consisting of PC and PE.

Differences in phospholipid metabolism between PC3 cells in culture and in subcutaneous tumours derived from these cells have been previously reported and was attributed to the influence of the tumour microenvironment on choline and lipid metabolism.^47^ However, our MRS detected phospholipid changes observed in the colorectal HT29 subcutaneous tumours are consistent with our in vitro findings both in HT29 and PC3 cells and also in line with the previously published MRS changes using the AKT inhibitors perifosine and MK-2206 in breast cancer cells.^27,29^ We also did not observe any differences in the cellular or molecular effects of MK-2206 in both tumour models. We therefore questioned whether the difference in the phospholipid biomarker changes in the PC3 subcutaneous tumours was due to the location of the tumour, and whether growing PC3 tumours orthotopically would result in a different metabolic response to treatment with the AKT inhibitor MK-2206 compared to PC3 subcutaneous tumours. Orthotopic tumour models are more clinically relevant compared to subcutaneous tumours. A previous study showed images in real time, using green fluorescent protein expression, of the very different tumour behaviour at the orthotopic and subcutaneous sites of human prostate cancer PC3 in athymic nude mice. The orthotopic tumour described had higher rates of vascularisation, migration, angiogenesis and metastasis compared to the subcutaneous tumour.^48^ Indeed, inhibition of AKT signalling with MK-2206 in orthotopic PC3 tumours resulted in a significant reduction in the tCho/water ratio using in vivo ^1^H-MRS and this was due to a decrease in PC levels as shown in the MRS analysis of the tumour extracts. Similar to MK-2206 treated PC3 and HT29 cells, orthotopic PC3 tumours treated with MK-2206 also showed reduced alanine and increased glucose, suggesting an alteration in glucose metabolism. MK-2206 had no effect on tumour bioenergetics, glutamine or glutathione metabolism, microvessel density, necrosis, proliferation or apoptosis. This shows that the difference in metabolic response between subcutaneous and orthotopic PC3 tumours could reflect the difference in tumour microenvironment at different tumour sites.

We have provided further evidence that in vitro inhibition of AKT is associated with changes in glucose, glutamine and choline metabolism both in prostate and colorectal cancer cell lines. We also demonstrated that the reduction in choline metabolites can be detected *in vivo* both in subcutaneous and the clinically relevant orthotopic prostate cancer tumours. A Phase I trial study published previously investigated the utility of ^1^H-MRS (amongst a number of functional imaging biomarkers) to monitor patient response to MK-2206.^7^ Individual but not cohort ^1^H-MRS detected changes in tCho/water ratio have been reported. This was possibly due to insufficient target and pathway modulation as the ultimate maximum tolerated dose was limited by dose limiting toxicities of rash during dose escalation. The Phase I study also involved a very small population of patients. The authors suggested that functional imaging studies including total choline levels should be considered in phase II trials using a higher dose of MK-2206.

Taken together, our MRS-detected choline metabolites may have potential as non-invasive biomarkers for monitoring response to treatment with AKT inhibitors during Phase I/II clinical trials in selected cancer types.

## Electronic supplementary material

Supplementary Information

## Availability of data and materials

All data generated or analysed during this study are included in this published article 603 and its supplementary information files.
