# Supplementary material for: Metabolic biomarkers of response to the AKT inhibitor MK-2206 in pre-clinical models of human colorectal and prostate carcinoma
Source: Br J Cancer. 2018 Oct 31;119(9):1118–28. doi: 10.1038/s41416-018-0242-3 (PMC6219501; doi:10.1038/s41416-018-0242-3)
Supplement: Supplementary file 1 — Supplementary Information [file 41416_2018_242_MOESM1_ESM.docx]

**Supplementary Figure S1.** (**A**) Representative immunoblots showing changes in molecular markers demonstrating AKT inhibition and absence of apoptosis, following treatment with MK-2206 (3xGI_50_, 24 h). β-Actin is used as a loading control. A summary of ^1^H-MRS metabolic changes caused by 24 h treatment of PC3 prostate cancer cells with MK-2206 at 3xGI_50_ vs. 5xGI_50_: (**B**) Choline–containing metabolites. (**C**) Glycolytic intermediates (Lac = lactate; Gluc = glucose), amino acids (Ala = alanine; Glu = glutamate; Gln = glutamine; GSH = glutathione) and PCr/Cr. Results are expressed as %T/C and presented as mean ± SD, n ≥ 4. Statistically significant differences from the control ^*^*P* ≤ 0.05, ^**^*P* ≤ 0.01; ^***^*P* ≤ 0.001.





**Supplementary Figure S2.** Molecular and metabolic changes caused by MK-2206 (5xGI_50_, 24 h) in HT29 colorectal cancer cells. (**A**) Representative immunoblots showing changes in molecular markers demonstrating AKT inhibition and absence of apoptosis. β-Actin is used as a loading control. (**B**) Flow cytometry analysis showing cell cycle distribution of cells with vehicle treatment (DMSO, control), or following treatment with MK-2206, *P* < 0.01 for G_1_, S& G_2_ phases. (**C**) Representative *in vitro* ^31^P-MR spectra (left) and expansion of ^1^H-MR spectra regions (1.3 - 3.3 ppm; right) showing choline–containing metabolites, Cr/PCr, Lac = lactate and amino acids (Ala = alanine; Glu = glutamate; Gln = glutamine; GSH = glutathione). A summary of ^1^H-MRS metabolic changes caused by treatment with MK-2206: (**D**) Choline-containing metabolites. (**E**) Amino acids, Cr/PCr and glycolytic intermediates (Gluc = glucose). Results are expressed as %T/C and presented as mean ± SD (error bars), n = 6. Statistically significant differences from the control ^*^*P* ≤ 0.05, ^**^*P* ≤ 0.01; ^***^*P* ≤ 0.001.





**Supplementary Figure S3.** Representative Western blots showing changes in protein expression levels of enzymes involved in phospholipid (CHKA) and glucose (HK2 and LDHA) metabolism post-treatment relative to controls in: (A) PC3 or (B) HT29 cells. β-Actin is used as a loading control.





**Supplementary Figure S4.** A summary of ^1^H-MRS metabolic changes detected in growth media following treatment with MK-2206 in (**A**) PC3. (**B**) HT29 cells (Lac = lactate; Ala = alanine; Glu = glutamate; Gln = glutamine; cho = choline; Gluc = glucose). Results are expressed as %T/C and presented as mean ± SD, n = 3. Statistically significant differences from the control ^*^*P* ≤ 0.05, ^**^*P* ≤ 0.01; ^***^*P* ≤ 0.001.





**Supplementary Figure S5.** MSD measurements of pP70S6K (Thr421/Ser424), total P70S6K, pRPS6 (Ser235/236), total RPS6, pAKT (Ser473), pAKT (Thr308) and total pAKT, in MK-2206 and vehicle-treated HT29 tumour lysates. Data are expressed as mean ± SEM. **P* < 0.05 and ****P* < 0.001, when comparing MK-2206 treated with vehicle controls, two-tailed unpaired *t*-test was used for all comparisons. Minimum n = 3.





**Supplementary Figure S6.** MSD measurements of pP70S6K (Thr421/Ser424), total P70S6K, pRPS6 (Ser235/236), total RPS6, pAKT (Ser473), pAKT (Thr308) and total AKT, in MK-2206 and vehicle-treated subcutaneous PC3 tumour lysates. Data are expressed as mean ± SEM. ***P* < 0.01, ****P* < 0.001 and *****P* < 0.0001, when comparing MK-2206 treated with vehicle controls, two-tailed unpaired *t*-test was used for all comparisons. Minimum n = 5.





**Supplementary Figure S7.** MSD measurements of pRPS6 (Ser240/244), total RPS6, pAKT (Ser473), pAKT (Thr308) and total AKT, in MK-2206 and vehicle-treated orthotopic PC3 tumour lysates. Data are expressed as mean ± SEM. ***P* < 0.01 and *****P* < 0.0001, when comparing MK-2206 treated with vehicle controls, two-tailed unpaired *t*-test was used for all comparisons. n = 6.
